# Immune related gene expression in worker honey bee (*Apis mellifera carnica*) pupae exposed to neonicotinoid thiamethoxam and *Varroa* mites (*Varroa destructor*)

**DOI:** 10.1371/journal.pone.0187079

**Published:** 2017-10-31

**Authors:** Tanja Tesovnik, Ivanka Cizelj, Minja Zorc, Manuela Čitar, Janko Božič, Gordana Glavan, Mojca Narat

**Affiliations:** 1 Department of Animal Sciences, Biotechnical Faculty, University of Ljubljana, Domžale, Slovenia; 2 Department of Biology, Biotechnical Faculty, University of Ljubljana, Ljubljana, Slovenia; University of North Carolina at Greensboro, UNITED STATES

## Abstract

*Varroa destructor* is one of the most common parasites of honey bee colonies and is considered as a possible co-factor for honey bee decline. At the same time, the use of pesticides in intensive agriculture is still the most effective method of pest control. There is limited information about the effects of pesticide exposure on parasitized honey bees. Larval ingestion of certain pesticides could have effects on honey bee immune defense mechanisms, development and metabolic pathways. Europe and America face the disturbing phenomenon of the disappearance of honey bee colonies, termed Colony Collapse Disorder (CCD). One reason discussed is the possible suppression of honey bee immune system as a consequence of prolonged exposure to chemicals. In this study, the effects of the neonicotinoid thiamethoxam on honey bee, *Apis mellifera carnica*, pupae infested with *Varroa destructor* mites were analyzed at the molecular level. *Varroa*-infested and non-infested honey bee colonies received protein cakes with or without thiamethoxam. Nurse bees used these cakes as a feed for developing larvae. Samples of white-eyed and brown-eyed pupae were collected. Expression of 17 immune-related genes was analyzed by real-time PCR. Relative gene expression in samples exposed only to *Varroa* or to thiamethoxam or simultaneously to both *Varroa* and thiamethoxam was compared. The impact from the consumption of thiamethoxam during the larval stage on honey bee immune related gene expression in *Varroa*-infested white-eyed pupae was reflected as down-regulation of *spaetzle*, AMPs *abaecin* and *defensin-1* and up-regulation of *lysozyme-2*. In brown-eyed pupae up-regulation of *PPOact*, *spaetzle*, *hopscotch* and *basket* genes was detected. Moreover, we observed a major difference in immune response to *Varroa* infestation between white-eyed pupae and brown-eyed pupae. The majority of tested immune-related genes were upregulated only in brown-eyed pupae, while in white-eyed pupae they were downregulated.

## Introduction

Pollinators are crucial in almost all terrestrial ecosystems, especially in those dominated by agriculture. Among pollinators in Slovenia the most important is the Carniolan honey bee, *Apis mellifera carnica* [1]. Unfortunately, high mortality rates in overwintering colonies have been documented over the last few years in North America and Europe [2, 3]. Besides the impact this has on ecological balance, such declines also have a great economic impact. Due to these massive losses, multiple candidate factors have been studied, including exposure to pesticides and pathogens [3].

The role of pesticides in honey bee losses, and their sub-lethal and synergistic impact, have been the subject of an increasing number of studies. Adult worker honey bees are repeatedly exposed to pesticides during the collection of pollen and nectar. By feeding developing honey bees contaminated food, entire colonies can be exposed to pesticides [4, 5]. Such sub-lethal exposure to pesticides can influence their physiology and behavior [6] and can cause changes in immune response and detoxification mechanisms [7–9].

In particular, neonicotinoid pesticides are currently receiving special attention [10]. These insecticides are chemically similar to nicotine, thus they act antagonistically to insect nicotine acetylcholine receptors [11]. Consequently, neonicotinoids may reduce the learning performance of honey bees, making them more susceptible to pathogens and other factors that result in colony loss [12, 13]. The neonicotinoid thiamethoxam is commonly used in agriculture and has been found as residue in honey bee colonies [4]. Recent studies demonstrated that even the lowest exposure dose of thiamethoxam elicits sub-lethal deleterious effects in adult honey bees [14] and toxicity of thiamethoxam on honey bee larvae [15]. In addition to pesticides, pathogenic infections can also contribute to colony mortality [1, 7].

*Varroa destructor* is one of the most widespread honey bee parasites and vector of viral and other honey bee pathogens [16]. This ectoparasitic mite is one of rare adult honey bee parasite that also affects brood [17]. Several studies [9, 16, 18–22] show that *Varroa* mites influence immune response, but results are contradictory. It has been suggested that *Varroa* parasitism suppresses honey bee immunity leaving them vulnerable to a variety of viruses [18–23]. While other studies of *Varroa* infestation in developing stages of worker bees showed increased expression of immune-related genes [9, 16, 24, 25]. As findings are hard to interpret, we are still far from understanding the interactions between the parasite and the honey bee immune system.

The immune system of honey bees possesses obvious orthologues for the major members of the immune pathways consisting of: Toll (transmembrane signal transducing pathway), Imd (immune deficiency), JNK (intracellular signaling pathways) and JAK/STAT (Janus kinase/signal transducers and activators of transcription). Although there have been several studies on honey bee immune responses to pathogens in combination with pesticides [7, 9, 16, 24], the effect of thiamethoxam in combination with *Varroa* infestation on the immune system has not yet been studied. The present study focuses on the effect of thiamethoxam and *Varroa* mites on honey bee immune-related gene expression in two developmental stages (white-eyed and brown-eyed pupae). According to previous studies describing the ontogeny of honey bee immune system [17, 26] our hypothesis was that thiamethoxam consumed in larval stage will have an influence on *Varroa* induced immune response in pupae.

## Material and methods

The honey bees used in this study were from *Apis mellifera carnica* colonies maintained at the Biotechnical Faculty, University of Ljubljana, Slovenia. Experiments were conducted in the Neuroethology Laboratory of the Biotechnical Faculty, University of Ljubljana. Colonies did not present visible symptoms of any known brood disease, e.g. American foulbrood, European foulbrood, Chalkbrood, or Stonebrood, which was also confirmed with RT-qPCR.

### Experimental setup

Eight mixed honeybee colonies were established from two different honey bee colonies A and B. This was accomplished in two steps. First, the two colonies were mixed, and separated into two hives, original queens were added to each colony. One colony was deliberately infested with *Varroa* mites by the addition of two brood frames from a heavily *Varroa* infested colony (colony C; more than 5% of 50 randomly sampled pupae infested). *Varroa* mite infestation was determined by brood sampling. Honey bee workers from both original colonies, A and B, were distributed, approximately equally, to the newly established pre-experimental colonies. The aim of this step was to establish two pre-experimental colonies that were a balanced mixture of brood and adults from colonies A and B. Each pre-experimental colony was formed from two unsealed brood frames from original colony A and two unsealed brood frames from original colony B. In both cases two additional frames with honey was added. Two additional frames from the colony C were not used to setup colonies in the next steps. After 10 days, each brood frame from pre-experimental colonies A and B were split into a separate nucs, establishing eight starter colonies for two biological repetitions. To increase survival of the experimental colonies, an additional frame of unsealed brood from an original colony, either from A or B, was added to each experimental colony. Finally every experimental colony had one experimental frame and one “helper” frame of the brood and additional frame for feeding with prepared protein sugar patties. To ensure normal function of all colonies, an unfertilized queen of the same origin was added to each colony. Queens have successfully mated until the treatment procedure (see below) and they started laying eggs. The infested and non-infested colonies were placed 5–6 m apart inside of tide bushy area to reduce chance for reinvasion (drifting) during the experiment. The treatment procedure started immediately afterwards (day 0).

Each day the colonies were fed with a 50 g protein cake (4 g chicken egg yolk, 8 g yeast, 13 g tofu, 72 g powdered sugar in 1 ml water and 2 g olive oil; all ingredients were the products of organic farming). Four colonies, two infested with *Varroa* mites and two non-infested, received protein cake with thiamethoxam (10 μg/kg cake; Actara®, Syngenta). The concentration of thiamethoxam in the cake was chosen according to Pilling et al. [27] where evaluated residues of thiamethoxam in pollen collected from honey bees after foraging on flowering seed treated maize were between 1 and 7 μg/kg. Nurse bees collect small portions of protein cake and mix it with honey. This mixture is then normally stored for a short time in the nurse bee crops only for the purpose of the delivery to the larvae and it is not transported to the midgut where the digestion is taking place involving a considering amount of enzymes [28]. To the other four honey bee colonies, two infested with *Varroa* mites and two non-infested, cakes without thiamethoxam were provided. Freshly made cakes were replaced every second day and the consumption of the cake was recorded. On day ten, capped brood cells were opened and 12 white-eyed pupae and 12 brown-eyed stage worker pupae at the start of cuticle pigmentation were collected from each colony. According to COLOSS BEEBOOK [29] white-eyed pupae represent a developmental stage of 0–5 days after cell capping and brown-eyed pupae 8–12 days after the capping. In the colonies that were deliberately infested with *Varroa* mites only parasitized pupae were sampled. A pupa was considered to be parasitized if a reproductive *Varroa* was found inside the brood cell together with the pupa. From the non-infested colonies only the pupae without *Varroa* mites were used for further analysis. The samples were collected and snap frozen using N_2_ and stored at -80°C until RNA extraction. The set-up of treatments is summarized in S1 Table.

### RNA isolation and cDNA synthesis

Individual white- or brown-eyed pupae were ground in liquid nitrogen and total RNA was extracted using TRIZOL® (Ambion®, Life Technologies) and then purified with PureLink® RNA Mini Kit columns (Ambion®, Life Technologies). RNA concentrations were determined using NanoVue (GE Healthcare, Waukesha, USA) and eight samples of white- or brown-eyed pupae from each biological group with best quality RNA were selected for further analysis. Residual DNA was removed by incubating 1 μg of RNA with 1 U of RNase-free DNase I (Fermentas, Germany) for 30 min at 37°C. High-Capacity cDNA Reverse Transcription Kit (Applied Biosystems, USA) was used to synthesize complementary DNA (cDNA), according to the manufacturer's instructions.

### Real-time PCR

Primer pairs used for candidate genes (related to immune response traits) were those reported in Gregorc et al. [9] and Evans [30] (Table 1). For quantitative real-time PCR, 10 μL reactions were prepared, containing 5 μL of Fast Start Universal SYBR Green Master (ROX) (Roche Diagnostics GmbH, Germany), 250 nM of forward and reverse primer, DEPC treated water and 5 ng of cDNA. Amplification of targets was performed with ViiA7 System (Applied Biosystems, USA) and analyzed with QuantStudio™ Real-Time PCR Software. For the experimental run the following cycle profile was used: first cycle at 95°C for 10 min and 40 cycles at 95°C for 20 s, 20 s at Tm of each primer pair and 72°C for 20s, followed by last cycle for 15 s at 95°C, 60s at Tm and 15 s at 95°C for dissociation curve. Reactions for quantitative real-time PCR were carried out in 384-well plates (MicroAmp®, Life Technologies). Each experiment contained three no-template controls and test samples performed in duplicates. Gene expression was analyzed for 17 immune-related genes. As reference genes we used *Rp49*, *RPS5* and *Tbp-af* according to its stable expression in our and previous studies [8, 31]. Gene expression values of non-treated group were used for gene expression calibration. For each gene the level of gene expression was calculated using the method described by Pfaffl, where the relative expression ratio between treated and non-treated group is based on PCR efficiency [32]. The results demonstrating the level of gene expression are shown in log_2_ scale. All collected samples were also tested for the most common honey bee-pathogen targets, including DWV, using RT-qPCR, performed as described before [8]. Gene expression was assayed for 10 honey bee-pathogen targets (S2 Table). The expression of pathogen genes of all the experimental samples were evaluated by comparing threshold cycle (CT) values between treatment groups.

**Table 1:** Housekeeping and immune-related primers used in this study.

Locus (gene ID)	Category	Gene description	F. Primer	R. Primer	R^2^	Efficiency (%)
**Rp49 (AF441189)**	**House keeping**	**Ribosomal protein 49**	**CGTCATATGTTGCCAACTGGT**	**TTGAGCACGTTCAACAATGG**	**0.997**	**100.2**
**RPS5 (GB11132)**	**House keeping**	**Ribosomal protein S5a**	**AATTATTTGGTCGCTGGAATTG**	**TAACGTCCAGCAGAATGTGGTA**	**0.997**	**100.8**
**Tbp-af (XM_393492)**	**House keeping**	**TATA box binding protein (TBP)—associated factor**	**TTGGTTTCATTAGCTGCACAA**	**ACTGCGGGAGTCAAATCTTC**	**0.997**	**99.9**
**Abaecin (GB18323)**	**Immune**	**Abaecin, antimicrobial peptide**	**CAGCATTCGCATACGTACCA**	**GACCAGGAAACGTTGGAAAC**	**0.999**	**99.8**
**Basket (GB16401)**	**Immune**	**JNK MAP kinase**	**AGGAGAACGTGGACATTTGG**	**AATCCGATGGAAACAGAACG**	**0.980**	**96.5**
**Cactus (GB13520)**	**Immune**	**IkB transcription factor**	**TGGTTGTCGTGCCAATACAG**	**TGTTCCAGTGAAAACGCAAT**	**0.993**	**99.9**
**Defensin1 (GB19392)**	**Immune**	**Defensin, antimicrobial peptide**	**TGCGCTGCTAACTGTCTCAG**	**AATGGCACTTAACCGAAACG**	**0.993**	**99.0**
**Defensin2 (GB10036)**	**Immune**	**Defensin 2, antimicrobial peptide**	**GCAACTACCGCCTTTACGTC**	**GGGTAACGTGCGACGTTTTA**	**0.991**	**98.9**
**Domeless (GB16422)**	**Immune**	**Cytokine receptor; JAK-STAT immune signaling pathway**	**TTGTGCTCCTGAAAATGCTG**	**AACCTCCAAATCGCTCTGTG**	**0.960**	**97.2**
**Dorsal-1 (GB19537)**	**Immune**	**NFkB transcription factor orthologue**	**AGAGATGGAACGCAGGAAAC**	**TGACAGGATATAGGACGAGGTAA**	**0.994**	**99.7**
**Dredd (GB17683)**	**Immune**	**Caspase-8**	**GCGTCATAAAGAAAAAGGATCA**	**TTTCGGGTAATTGAGCAACG**	**0.955**	**102.2**
**Hopscotch (GB12159)**	**Immune**	**JAK tyrosine kinase**	**ATTCATGGCATCGTGAACAA**	**CTGTGGTGGAGTTGTTGGTG**	**0.978**	**100.4**
**lap2 (XM_396819.5)**	**Immune/ apoptosis**	**Inhibitor of apoptosis 2**	**ACCTTGCCAAAGCTGGAT**	**TCATCACCAAGCTCCCATTT**	**0.987**	**101.2**
**Kayak (GB12212)**	**Immune**	**Fos, Drosophila homologue of the mammalian proto-oncogene product c-Fos**	**CGACAGATCCGCAGAGAAAG**	**CCTGTTGCAGCTGTTGTATC**	**0.990**	**101.8**
**Lys2 (GB15106)**	**Immune**	**Lysozyme, immune end product**	**CCAAATTAACAGCGCCAAGT**	**GCAATTCTTCACCCAACCAT**	**0.990**	**97.8**
**PGRPLC710 (GB17188)**	**Immune**	**Peptidoglycan recognition protein LC**	**TCCGTCAGCCGTAGTTTTTC**	**CGTTTGTGCAAATCGAACAT**	**0.979**	**95.6**
**PGRPSC4300 (GB15371)**	**Immune**	**Peptidoglycan recognition protein S1**	**GAGGCTGGTACGACATTGGT**	**TTATAACCAGGTGCGTGTGC**	**0.996**	**100.2**
**PPOact (GB18767)**	**Immune**	**Serine protease 8**	**GTTTGGTCGACGGAAGAAAA**	**CCGTCGACTCGAAATCGTAT**	**0.989**	**101.8**
**Spaetzle (GB15688)**	**Immune**	**Toll-binding cytokine-like molecule**	**TGCACAAATTGTTTTTCCTGA**	**GTCGTCCATGAAATCGATCC**	**0.989**	**95.5**
**Toll (GB18520)**	**Immune**	**Toll-like receptor**	**TAGAGTGGCGCATTGTCAAG**	**ATCGCAATTTGTCCCAAAAC**	**0.977**	**100.6**

### Statistical analysis

To analyze the effects of *Varroa* mite infestation, thiamethoxam treatment and their interaction on gene expression, we used linear model for fixed effects. The estimation of least squares means followed by Dunnett’s post hoc test was used for pairwise comparisons among the treatment groups. The assumption of normal distribution was tested and met via examination of the residuals (coefficients of skewness and kurtosis). Differences between levels were considered to be statistically significant if the p-value was equal to or less than 0.05. All statistical analyses and plotting were carried out using R software version 3.4.1 [33] with relevant libraries (lsmeans, moments, ggplot2) [34–36].

### Pathway analysis of honey bee immune gene expression data

PathVisio, the graphical editor for biological pathways [37], was used for visualization of honey bee immune pathways. We have used the WikiPathways plugin for PathVisio to search for immune pathways in insects in the online pathway database. As a base, we used the *Drosophila melanogaster* pathway, (WP3830—Toll, IMD, and JAK/STAT Pathways for Immune Response to Pathogens). We have modified selected pathway diagrams: 1) all the elements presenting genes which were not annotated in the *Apis mellifera* genome (version Amel_4.5) were removed, 2) JNK pathway was added, 3) effector genes, such as *defensin1*, *defensin2*, and *abaecin* were added. Thus we developed honey bee specific Toll, IMD, JAK/STAT and JNK pathway diagrams.

The honey bee pathway diagram was used for differentially expressed immune-related genes analysis. Using the ggplot library for R, we plotted a heatmap (S1 Fig), where colors indicate the average mRNA levels of *Varroa* treated groups compared to average levels of mRNA in groups simultaneously treated with thiamethoxam and infested with *Varroa*. We mapped the colors from the scale of the heatmap to the elements of pathway diagrams (S1 Fig).

## Results

### Immune-related gene expression in white-eyed pupae

**Fig 1:**
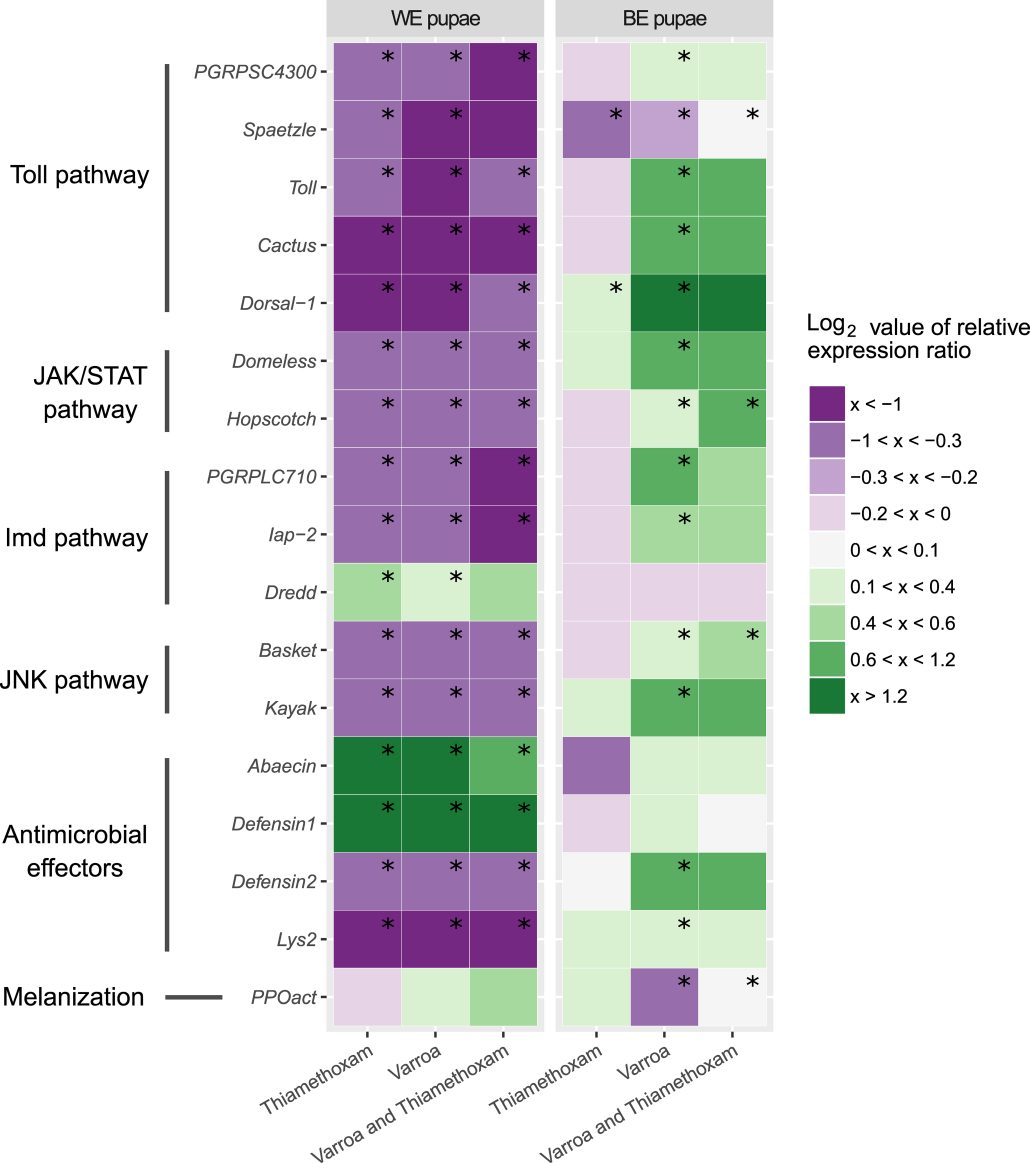
Heatmap of differential expression of immune-related genes in white-eyed and brown-eyed pupae. The colors indicate the average mRNA levels compared to average levels of mRNA in control groups: purple indicates lower and green higher levels. Range log_2_ value of relative expression ratio is indicated in the legend on the right. Each column corresponds to the expression profile of one treatment (Thiamethoxam; *Varroa*; Thiamethoxam and *Varroa*) and each row, to one gene transcript. The immune-related gene names and corresponding immune pathway are indicated on the left. Boxes marked with symbol (*) shows statistically significant effect of treatment on gene expression if the p-value was equal to or less than 0.05.

**Fig 2:**
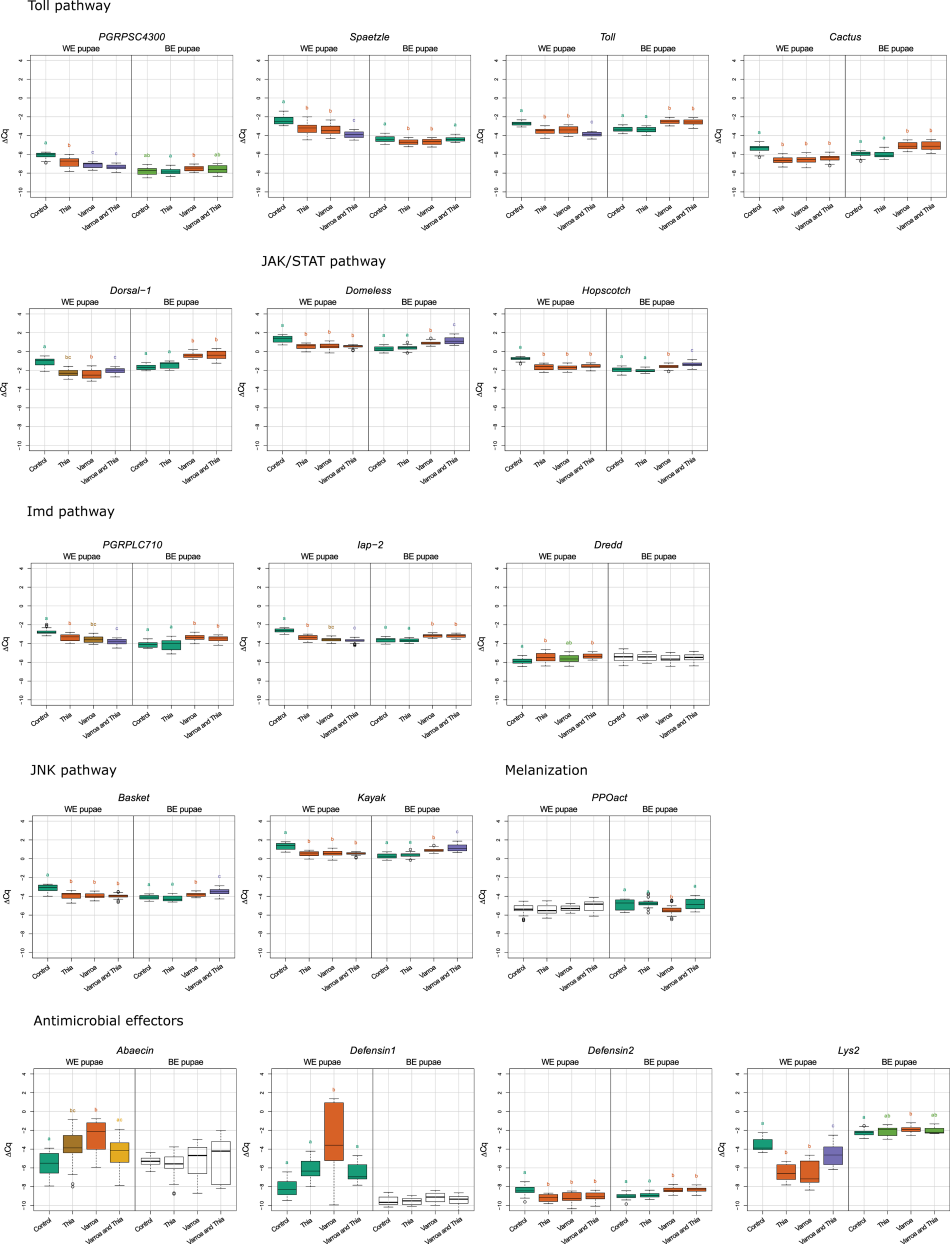
Box plot diagrams for immune-related gene expression. Each box plot represents the ΔCq values measured for biological replicates for selected treatment and developmental stage. Treatments sharing the same letter are not significantly different from one another within the same developmental stage. Treatments are indicated in the scale at the bottom of the plots: Control; Thia: thiamethoxam; *Varroa*; *Varroa* and Thia: *Varroa* and thiamethoxam. Analysis was undertaken with program R. The results demonstrating the level of gene expression are shown in log_2_ scale.

In white-eyed pupae, only four genes were upregulated in at least one treatment, while 13 immune-related genes were downregulated in all cases. Thiamethoxam significantly upregulated genes *defensin-1* (3.94), *abaecin* (3.26) and *dredd* (1.38), while 13 genes were significantly downregulated. Among the most downregulated were *lysozyme-2* (0.13), *cactus* (0.44) and *dorsal-1* (0.44) (Fig 1). In *Varroa* infested groups, *abaecin* (6.30), *dredd* (1.24) and *defensin-1* (30.53) had statistically significant upregulation while the most downregulated genes were *lysozyme-2* (0.13) and *dorsal-1* (0.40) (Fig 2). In groups simultaneously exposed to thiamethoxam and infested with *Varroa*, we can see a similar gene expression pattern as in the other two groups, but upregulation of *dreed* was not statistically significant. Regardless of treatment, all white-eyed pupae transmembrane proteins were downregulated, and no immune-related pathways were activated, except part of the Imd pathway (*dredd*). The only effector genes that were upregulated were *abaecin* and *defensin-1* (Fig 1).

### Immune-related gene expression in brown-eyed pupae

In brown-eyed pupae, thiamethoxam caused mild effects in gene expression resulting in statistically significant downregulation of only *spaetzle* (Fig 1). In *Varroa* infested brown-eyed pupae, gene expression pattern was completely changed compared to white-eyed pupae. The majority of genes tested were upregulated and only two genes, *PPOact* and *spaetzle*, were downregulated (Fig 1). The highest statistically significant upregulation was detected for *dorsal-*1 (2.39). In *Varroa* infested pupae, thiamethoxam treatment significantly changed the expression of ten genes (Fig 2). The pathway diagrams show that all transmembrane proteins are upregulated, which resulted in activation of all four immune-related pathways (Toll, JAK/STAT, IMD and JNK) and effector genes (*defensin-2*, *lysozyme-2*, and *PPOact*) (Fig 1).

### The difference in the mean expression of each gene between groups infested with *Varroa* and group simultaneously infested with *Varroa* and treated with thiamethoxam

**Fig 3:**
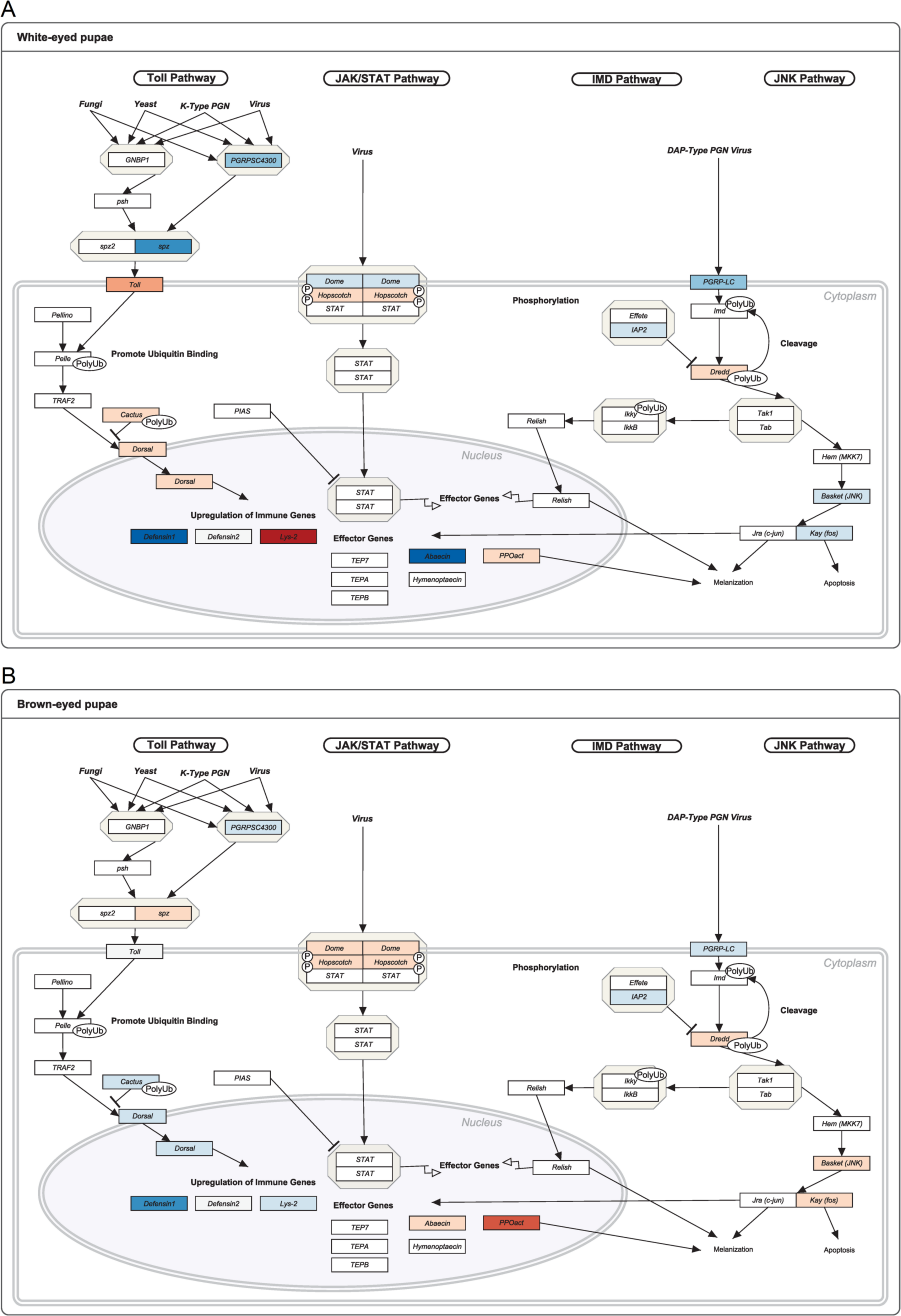
The effect of thiamethoxam on immune related gene expression in *Varroa* infested bees. The expression level for each gene is presented as difference in average expression level in *Varroa* infested group and infested-thiamethoxam treated group. The higher expression levels are indicated with red color, lower expression levels are indicated with blue color. Immune pathway diagram for white-eyed pupae (A) and brown-eyed pupae (B) are presented. The range of relative expression ratio is indicated in the legend (S1 Fig). Bluer color in panel indicates higher influence of thiamethoxam on *Varroa* infested pupae.

We compared differences in the mean expression of each gene between *Varroa*-infested colonies and those that were *Varroa*-infested and treated with thiamethoxam. In infested white-eyed pupae we observed that consumption of thiamethoxam in larval stage led to downregulation of *spaetzle*, *abaecin* and *defensin-1*, but it also caused higher expression of gene *lysozyme-2* (Fig 3A). Expression of *spaetzle*, *hopscotch*, *basket* and *PPOact* genes was statistically increased in brown-eyed pupae with addition of thiamethoxam to *Varroa* infested group (Fig 3B). The only gene on which thiamethoxam had no effect in both white- and brown-eyed infested pupae was *defensin-2*.

## Discussion

The strength of honey bee colony strongly depends on the brood so studies regarding the effects of various neonicotinoids on larvae should be performed [10]. It has been revealed that even sub-lethal concentrations of thiamethoxam can affect the larval development of honey bee [15]. In different studies the effect of neonicotinoids and *Varroa* infestation has been studied but there is limited information about their combined effect. In our study, the majority of immune-related genes were downregulated in white-eyed pupae in all treatments. In *Varroa*-infested white-eyed pupae the only significantly upregulated genes were two antimicrobial effectors, *abaecin* and *defensin-1* (Fig 1). Antimicrobial peptides (AMPs) are evolutionary important components of insect immune systems. They can be activated and delivered in a short period of time to the site of infection [38]. Abaecin is a crucial immune effector involved in reactions against multiple parasites. Upregulation of several AMP genes, including *abaecin* and *defensin-1*, was also shown in other studies where honey bees were infested with mites [9, 25]. Even though in our case Toll pathway was downregulated, the expression of *abaecin* can be also regulated by Imd pathway, through *relish* [39]. Second AMP, upregulated in white-eyed pupae was *defensin-1*. Latest report showed that DWV could contribute to *defensin 1* upregulation [25] therefore we suggest that *defensin 1* could be modulated by DWV and different organisms that we may not have diagnosed, like bacteria, fungi, viruses and microsporidia. Defensin 1 is involved in the formation of social immunity, as peptide in royal jelly, by forming channels in plasma membrane that reduce integrity and permeability of cytoplasmic membrane in pathogenic organisms [38]. It appears that antimicrobial peptides, like Defensin 1, are able to protect honey bees even in early developmental stages and act as part of individual immunity [40]. Since *defensin-1* is not regulated through Imd pathway [39] and in our case Toll pathway is not activated either, our results suggest that *defensin-1* could be regulated through another pathway. In all the treatments down-regulation of *iap-2* gene was noticed. Inhibitor of apoptosis 2 is known to be important for sustained response in the Imd pathway and its inactivation resulted in impaired microbial resistance in *Drosophila* [41]. Interestingly, the only thiamethoxam-dependent upregulation was detected for Dredd, a cytoplasmic caspase, involved in activation of Imd pathway, through cleavage of Relish [42].

In both groups of brown-eyed pupae, *Varroa* infested and simultaneously infested and thiamethoxam treated, the majority of genes became upregulated. In these two groups the cascades of genes involved in all four pathways, Toll, JAK/STAT, Imd and JNK, were upregulated (Fig 1). It was reported [9, 26, 42] that peptidoglycan recognition proteins (PGRPs) are associated with mite parasitism and other pathogen infections. In our study, *Varroa* parasitism increased expression of *PGRP-SC 4300*, *PGRP-LC 710* and *domless*. Activation of these three genes leads to activation of genes involved in all four pathways including effectors genes, *defensin-2* and *lysozyme-2* [16]. Contrary to earlier studies [19, 20, 23] our data show little evidence for reduced expression of immune-related genes in *Varroa* infested brown-eyed pupae. The only statistically significant downregulation was detected for two genes, *spaetzle* and *PPOact*. Prophenoloxidases (PPOact) are key enzymes responsible for activation of melanin synthesis, important in defense against microbes and wounding. Expression of this gene plays a crucial role in inhibition of parasite development in insects [9, 43]. Spaetzle is known as a trigger for Toll pathway resulting in transcription of effectors (AMPs, *PPOact* and lysozyme) [26]. In brown-eyed pupae, thiamethoxam alone had only a minor effect on immune-related genes with no statistical significance.

The impact of in larval stage ingested thiamethoxam on *Varroa* infested white-eyed and brown-eyed pupae, had a significant effect that could be seen on heatmap (Fig 1) only when the mean expression level of each gene between *Varroa* infested and *Varroa* infested-thiamethoxam treated groups were compared (Fig 2). Normally, each treated group only to control group is compared.

In infested white-eyed pupae, consumption of thiamethoxam leads to even stronger downregulation of *spaetzle*, *abaecin* and *defensin-1*, but it also caused higher expression of gene *lysozyme-2*, *toll*, and *dorsal-1* when compared to group infested with *Varroa* (Figs 2 and 3B). It can be presumed that larval exposure to thiamethoxam deteriorates defense against *Varroa* mites and *Varroa*-mite associated viruses in pupal development stage. In brown-eyed pupae consumption of thiamethoxam to *Varroa* infested group leads to statistically significant increased expression of *spaetzle*, *hopscotch*, *basket*, and *PPOact* genes (Figs 2 and 3B). The only gene on which thiamethoxam had no effect in both white- and brown-eyed infested pupae was *defensin-2*. Expression of *defensin-2* is induced by a pathogenic factor, and plays a role in individual immunity. Thiamethoxam caused more decline in gene expression on infested white-eyed pupae than in brown-eyed pupae that develop 3–4 days later.

During the infestation *Varroa* mites can vector viruses and other pathogens within and between colonies. Moreover one of the consequences of *Varroa* parasitism is a decline in immune capacity which appears to induce the proliferation of viruses such as deformed wings virus in bees [22]. Comparable to other studies [9, 16, 21, 44, 45] a positive correlation between DWV and *Varroa* mites was found. We demonstrated that DWV RNA loads correlate with *Varroa* infestation in honey bee pupae (S1 Fig). Therefore, when talking about *Varroa* induced gene expression modifications, it would be important to investigate DWV impact on gene expression patterns [46–48].

Our data showed evidence for both down- and up-regulation of immune-related genes between two developmental stages of honey bees. Some of our results support studies reporting downregulation [18–22] other upregulation [9, 16, 25] of immune-related genes. It is difficult to explain such differential response in immune-related genes in two different pupal stages, however there is a link between the pupal development and immune response, a process of melanization. During the pupal period the intensity of melanization is rising with the development, in brown-eyed pupae this process being more intense than in white-eyed pupae. In immunity melanization is an immune effector mechanism involved in the killing different pathogens which involves a series of reactions to form a layer of melanin that surrounds and sequesters an invading pathogen [49]. Our results clearly show a difference in gene expression pattern between white-eyed and brown-eyed pupae, where significant change from downregulation to upregulation of almost all immune-related genes could be seen in the two to three days transformation. This study represents novel information about immune response to combined effect of thiamethoxam and *Varroa* parasitism. However, honey bee immune response depends also on post-transcriptional regulation as well as on post-translational regulation of gene expression and small differences in expression could reflect subtle modulation by a large number of factors acting in cascade [20]. Additionally, it is known that expression of genes can differ across developmental stages in different organisms [50]. Therefore, to paint a clear picture of pathogen and pesticide effects on honey bees each developmental stage should be analyzed.

In conclusion, our data provide novel insight into the genetic response of developing honey bees to exposure to thiamethoxam and *Varroa* parasitism. We demonstrated that *Varroa*-induced activation of four known immune pathways does not occur earlier than in the brown-eyed pupal stage, and when thiamethoxam was present the significant alternation in expression of only 1/3 of analyzed genes was noticed. Thiamethoxam alone at concentrations within the range detected in hives had down-regulatory effects on immune-related genes in white-eyed pupae while in brown-eyed pupae its effect is minimal. In larval stage consumed thiamethoxam has an important and variable influence on immune related gene expression in *Varroa*-infested white-eyed and brown-eyed pupae. In white-eyed pupae down-regulation of several immune-related genes suggests increased thiamethoxam immune-toxicity after *Varroa*-infestation. In thiamethoxam intoxicated brown-eyed pupae up-regulation of major immune related genes indicate increase of immune activity after *Varroa* infestation. Our data indicates that thiamethoxam can contribute to *Varroa* harmful effects on honey bee pupae.

## Supporting information

S1 Table Experimental honey bee groups. (DOCX)

S2 Table Primers used for detection of honey bee pathogens. (DOCX)

S1 Fig Deformed wings virus RNA loads in white- and brown-eyed pupae. Box-plots represents qPCR treshold cycle (CT) values for pathogen loads for different treatments in white-eyed (WE) and brown-eyed (BE) pupae. Treatments: control; thiamethoxam (Thia); *Varroa*; thiamethoxam and *Varroa* (*Varroa* and Thia). In each group (white-eyed pupae and brown-eyed pupae) means with different letters were considered to be statistically significant if the p-value was equal to or less than 0.05.
(DOCX)

S2 Fig Heatmap analysis of the differences in the mean expression of each gene between *Varroa* infested and *Varroa* infested—thiamethoxam treated group. WE pupae, white-eyed pupae; BE pupae, brown-eyed pupae. The colors indicate the average mRNA levels in *Varroa* infested—thiamethoxam treated group compared to average levels of mRNA in *Varroa* infested group: blue indicate lower and red higher levels of expression. Range of relative expression ratio is indicated in the legend on the right.
(DOCX)

## References

[1] Kevan PG. Pollinators as bioindicators of the state of the environment: species, activity and diversity. Agr Ecosyst Environ. 1999;74(1–3):373–93. doi: 10.1016/S0167-8809(99)00044-4

[2] Neumann P, Carreck NL. Honey bee colony losses. J Apicult Res. 2010;49(1):1–6. doi: 10.3896/Ibra.1.49.1.01

[3] vanEngelsdorp D, Evans JD, Saegerman C, Mullin C, Haubruge E, Nguyen BK, et al Colony Collapse Disorder: A descriptive study. Plos One. 2009;4(8). doi: ARTN e6481 doi: 10.1371/journal.pone.0006481 10.1371/journal.pone.0006481 PMC2715894 19649264

[4] Mullin CA, Frazier M, Frazier JL, Ashcraft S, Simonds R, vanEngelsdorp D, et al High Levels of miticides and agrochemicals in North American Apiaries: Implications for honey bee health. Plos One. 2010;5(3). doi: ARTN e9754 doi: 10.1371/journal.pone.0009754 10.1371/journal.pone.0009754 PMC2841636 20333298

[5] Villa S, Vighi M, Finizio A, Serini GB. Risk assessment for honeybees from pesticide-exposed pollen. Ecotoxicology. 2000;9(4):287–97. doi: 10.1023/A:1026522112328

[6] Desneux N, Decourtye A, Delpuech JM. The sublethal effects of pesticides on beneficial arthropods. Annu Rev Entomol. 2007;52:81–106. Epub 2006/07/18. doi: 10.1146/annurev.ento.52.110405.091440 16842032 10.1146/annurev.ento.52.110405.091440

[7] Boncristiani H, Underwood R, Schwarz R, Evans JD, Pettis J, vanEngelsdorp D. Direct effect of acaricides on pathogen loads and gene expression levels in honey bees *Apis mellifera*. J Insect Physiol. 2012;58(5):613–20. Epub 2012/01/04. doi: 10.1016/j.jinsphys.2011.12.011 22212860 10.1016/j.jinsphys.2011.12.011

[8] Cizelj I, Glavan G, Bozic J, Oven I, Mrak V, Narat M. Prochloraz and coumaphos induce different gene expression patterns in three developmental stages of the Carniolan honey bee (*Apis mellifera carnica* Pollmann). Pestic Biochem Physiol. 2016;128:68–75. Epub 2016/03/13. doi: 10.1016/j.pestbp.2015.09.015 26969442 10.1016/j.pestbp.2015.09.015

[9] Gregorc A, Evans JD, Scharf M, Ellis JD. Gene expression in honey bee (*Apis mellifera*) larvae exposed to pesticides and Varroa mites (*Varroa destructor*). J Insect Physiol. 2012;58(8):1042–9. doi: 10.1016/j.jinsphys.2012.03.015 22497859 10.1016/j.jinsphys.2012.03.015

[10] Blacquiere T, Smagghe G, Van Gestel CAM, Mommaerts V. Neonicotinoids in bees: a review on concentrations, side-effects and risk assessment. Ecotoxicology. 2012; 21(4): 973–992. doi: 10.1007/s10646-012-0863-x 22350105 10.1007/s10646-012-0863-x PMC3338325

[11] Nauen R, Ebbinghaus-Kintscher U, Salgado VL, Kaussmann M. Thiamethoxam is a neonicotinoid precursor converted to clothianidin in insects and plants. Pestic Biochem Physiol. 2003;76(2):55–69. doi: 10.1016/S0048-3575(03)00065-8

[12] Decourtye A, Lacassie E, Pham-Delegue MH. Learning performances of honeybees (*Apis mellifera* L) are differentially affected by imidacloprid according to the season. Pest Manag Sci. 2003;59(3):269–78. Epub 2003/03/18. doi: 10.1002/ps.631 12639043 10.1002/ps.631

[13] Doublet V, Labarussias M, de Miranda JR, Moritz RFA, Paxton RJ. Bees under stress: sublethal doses of a neonicotinoid pesticide and pathogens interact to elevate honey bee mortality across the life cycle. Environ Microbiol. 2015;17(4):969–83. doi: 10.1111/1462-2920.12426 25611325 10.1111/1462-2920.12426

[14] Badiou-Beneteau A, Carvalho SM, Brunet JL, Carvalho GA, Bulete A, Giroud B, et al Development of biomarkers of exposure to xenobiotics in the honey bee *Apis mellifera*: Application to the systemic insecticide thiamethoxam. Ecotox Environ Safe. 2012;82:22–31. doi: 10.1016/j.ecoenv.2012.05.005 10.1016/j.ecoenv.2012.05.005 22683234

[15] Tavares DA, Roat TC, Carvalho SM, Silva-Zacarin ECM, Malaspina O. In vitro effects of thiamethoxam on larvae of Africanized honey bee *Apis mellifera* (Hymenoptera: Apidae). Chemosphere. 2015;135:370–8. doi: 10.1016/j.chemosphere.2015.04.090 25985214 10.1016/j.chemosphere.2015.04.090

[16] Kuster RD, Boncristiani HF, Rueppell O. Immunogene and viral transcript dynamics during parasitic *Varroa destructor* mite infection of developing honey bee (*Apis mellifera*) pupae. J Exp Biol. 2014;217(10):1710–8. doi: 10.1242/jeb.097766 24829325 10.1242/jeb.097766

[17] Wilson-Rich N, Dres ST, Starks PT. The ontogeny of immunity: development of innate immune strength in the honey bee (*Apis mellifera*). J Insect Physiol. 2008;54(10–11):1392–9. Epub 2008/09/02. doi: 10.1016/j.jinsphys.2008.07.016 18761014 10.1016/j.jinsphys.2008.07.016

[18] Di Prisco G, Annoscia D, Margiotta M, Ferrara R, Varricchio P, Zanni V, et al A mutualistic symbiosis between a parasitic mite and a pathogenic virus undermines honey bee immunity and health. P Natl Acad Sci USA. 2016;113(12):3203–8. doi: 10.1073/pnas.1523515113 10.1073/pnas.1523515113 PMC4812730 26951652

[19] Gregory PG, Evans JD, Rinderer T, de Guzman L. Conditional immune-gene suppression of honeybees parasitized by Varroa mites. J Insect Sci. 2005;5. PubMed PMID: ISI:000228141900001. 10.1093/jis/5.1.7 PMC1283888 16299597

[20] Navajas M, Migeon A, Alaux C, Martin-Magniette ML, Robinson GE, Evans JD, et al Differential gene expression of the honey bee *Apis mellifera* associated with *Varroa destructor* infection. Bmc Genomics. 2008;9. doi: Artn 301 doi: 10.1186/1471-2164-9-301 10.1186/1471-2164-9-301 PMC2447852 18578863

[21] Nazzi F, Brown SP, Annoscia D, Del Piccolo F, Di Prisco G, Varricchio P, et al Synergistic parasite-pathogen interactions mediated by host immunity can drive the collapse of honeybee colonies. Plos Pathog. 2012;8(6). doi: ARTN e1002735 doi: 10.1371/journal.ppat.1002735 10.1371/journal.ppat.1002735 PMC3375299 22719246

[22] Yang XL, Cox-Foster DL. Impact of an ectoparasite on the immunity and pathology of an invertebrate: Evidence for host immunosuppression and viral amplification. P Natl Acad Sci USA. 2005;102(21):7470–5. doi: 10.1073/pnas.0501860102 10.1073/pnas.0501860102 PMC1140434 15897457

[23] Koleoglu G, Goodwin PH, Reyes-Quintana M, Hamiduzzaman MM, Guzman-Novoa E. Effect of *Varroa destructor*, wounding and Varroa homogenate on gene expression in brood and adult honey bees. Plos One. 2017;12(1). doi: ARTN e0169669 doi: 10.1371/journal.pone.0169669 10.1371/journal.pone.0169669 PMC5232351 28081188

[24] Aronstein K, Douglas A. Lessons Learned by the Managed Pollinator CAP: Impacts of Varroa parasitism on honey bee health. Am Bee J. 2012;152(8):789–90. PubMed PMID: ISI:00030672490002.

[25] Ryabov EV, Fannon JM, Moore JD, Wood GR, Evans DJ. The Iflaviruses Sacbrood virus and Deformed wing virus evoke different transcriptional responses in the honeybee which may facilitate their horizontal or vertical transmission. Peerj. 2016;4. doi: Artn E1591 doi: 10.7717/Peerj.1591 10.7717/peerj.1591 PMC4727977 26819848

[26] Evans JD, Aronstein K, Chen YP, Hetru C, Imler JL, Jiang H, et al Immune pathways and defence mechanisms in honey bees *Apis mellifera*. Insect Mol Biol. 2006;15(5):645–56. doi: 10.1111/j.1365-2583.2006.00682.x 17069638 10.1111/j.1365-2583.2006.00682.x PMC1847501

[27] Pilling E, Campbell P, Coulson M, Ruddle N, Tornier I. A four-year field program investigating long-term effects of repeated exposure of honey bee colonies to flowering crops treated with thiamethoxam. Plos One. 2013;8(10):e77193 doi: 10.1371/journal.pone.0077193 24194871 10.1371/journal.pone.0077193 PMC3806756

[28] Balasubramanyam MV. Role of different enzymes in nectar to honey transformations in indigenous rockbee, *Apis dorsata* F. J Chem Bio Phy Sci. 2014;4(1):361–368.

[29] Human H, Brodschneider R, Dietemann V, Dively G, Ellis JD, Forsgren E, et al Miscellaneous standard methods for *Apis mellifera* research. In Dietemann V, Ellis JD, Neumann P, editors. The COLOSS BEEBOOK, Volume I: Standard methods for *Apis mellifera* research. J Apicult Res. 2013;52(4):1–53. doi: 10.3896/ibra.1.52.4.10

[30] Evans JD. Beepath: An ordered quantitative-PCR array for exploring honey bee immunity and disease. J Invertebr Pathol. 2006;93(2):135–9. doi: 10.1016/j.jip.2006.04.004 16737710 10.1016/j.jip.2006.04.004

[31] Lourenco AP, Mackert A, Cristino AD, Simoes ZLP. Validation of reference genes for gene expression studies in the honey bee, *Apis mellifera*, by quantitative real-time RT-PCR. Apidologie. 2008;39(3):372–U33. doi: 10.1051/apido:2008015

[32] Pfaffl MW. A new mathematical model for relative quantification in real-time RT-PCR. Nucleic Acids Res. 2001;29(9). doi: ARTN e45. doi: 10.1093/nar/29.9.e45 10.1093/nar/29.9.e45 PMC55695 11328886

[33] R Core Team. R: A language and environment for statistical computing. R Foundation for Statistical Computing, Vienna, Austria. 2017. https://www.R-project.org

[34] Lenth RV. Least-Squares Means: The R Package lsmeans. 2016. 2016;69(1):33. Epub 2016-01-29. doi: 10.18637/jss.v069.i01

[35] Komsta L, Novomestky F. Moments: Moments, cumulants, skewness, kurtosis and related tests. R package version 0.14. 2015. https://CRAN.R-project.org/package=moments

[36] Wickham H. Ggplot2: elegant graphics for data analysis. New York: Springer; 2009, 212 p. p.

[37] Kutmon M, van Iersel MP, Bohler A, Kelder T, Nunes N, Pico AR, et al PathVisio 3: An extendable pathway analysis toolbox. Plos Comput Biol. 2015;11(2). doi: UNSP e1004085 doi: 10.1371/journal.pcbi.1004085 10.1371/journal.pcbi.1004085 PMC4338111 25706687

[38] Ilyasov RA, Gaifullina LR, Saltykova ES, Poskryakov AV, Nikolenko AG. Review of the expression of antimicrobial peptide Defensin in honey bees *Apis Mellifera* L. J Apic Sci. 2012;56(1):115–24. doi: 10.2478/v10289-012-0013-y

[39] Schluns H, Crozier RH. Relish regulates expression of antimicrobial peptide genes in the honeybee, *Apis mellifera*, shown by RNA interference. Insect Mol Biol. 2007;16(6):753–9. doi: 10.1111/j.1365-2583.2007.00768.x 18093004 10.1111/j.1365-2583.2007.00768.x

[40] Evans JD. Transcriptional immune responses by honey bee larvae during invasion by the bacterial pathogen, Paenibacillus larvae. J Invertebr Pathol. 2004;85(2):105–11. doi: 10.1016/j.jip.2004.02.004 15050840 10.1016/j.jip.2004.02.004

[41] Valanne S, Kleino A, Myllymaki H, Vuoristo J, Ramet M. Iap2 is required for a sustained response in the *Drosophila* Imd pathway. Dev Comp Immunol. 2007;31(10):991–1001. Epub 2007/03/09. doi: 10.1016/j.dci.2007.01.004 17343912 10.1016/j.dci.2007.01.004

[42] Aggarwal K, Silverman N. Positive and negative regulation of the *Drosophila* immune response. Bmb Rep. 2008;41(4):267–77. 18452646 10.5483/bmbrep.2008.41.4.267

[43] Wang Y, Hao HX, Qiu ZW, Xu WY, Zhang J, Zhou TL, et al Involvement of prophenoloxidases in the suppression of *Plasmodium yoelii* development by *Anopheles dirus*. Exp Parasitol. 2009;123(1):6–10. doi: 10.1016/j.exppara.2009.05.017 19540233 10.1016/j.exppara.2009.05.017

[44] Francis RM, Nielsen SL, Kryger P. Varroa-virus interaction in collapsing honey bee colonies. Plos One. 2013;8(3). doi: ARTN e57540 doi: 10.1371/journal.pone.0057540 10.1371/journal.pone.0057540 PMC3602523 23526946

[45] Rosenkranz P, Aumeier P, Ziegelmann B. Biology and control of *Varroa destructor*. J Invertebr Pathol. 2010;103 Suppl 1:S96–119. Epub 2009/11/17. doi: 10.1016/j.jip.2009.07.016 19909970 10.1016/j.jip.2009.07.016

[46] Chen YP, Siede R. Honey bee viruses. Adv Virus Res. 2007;70:33–80. Epub 2007/09/04. doi: 10.1016/S0065-3527(07)70002-7 17765703 10.1016/S0065-3527(07)70002-7

[47] Shen MQ, Yang XL, Cox-Foster D, Cui LW. The role of varroa mites in infections of Kashmir bee virus (KBV) and deformed wing virus (DWV) in honey bees. Virology. 2005;342(1):141–9. doi: 10.1016/j.virol.2005.07.012 16109435 10.1016/j.virol.2005.07.012

[48] Yue C, Genersch E. RT-PCR analysis of Deformed wing virus in honeybees (*Apis mellifera*) and mites (*Varroa destructor*). J Gen Virol. 2005;86:3419–24. doi: 10.1099/vir.0.81401-0 16298989 10.1099/vir.0.81401-0

[49] Hillyer JF. Insect immunology and hematopoiesis. Dev Comp Immunol. 2016;58:102–18. doi: 10.1016/j.dci.2015.12.006 26695127 10.1016/j.dci.2015.12.006 PMC4775421

[50] Jefferson JM, Dolstad HA, Sivalingam MD, Snow JW. Barrier Immune effectors are maintained during transition from nurse to forager in the honey bee. Plos One. 2013;8(1). doi: ARTN e54097 doi: 10.1371/journal.pone.0054097 10.1371/journal.pone.0054097 PMC3540063 23320121

