# Supplementary material for: Immune related gene expression in worker honey bee (Apis mellifera carnica) pupae exposed to neonicotinoid thiamethoxam and Varroa mites (Varroa destructor)
Source: PLoS One. 2017 Oct 31;12(10):e0187079. doi: 10.1371/journal.pone.0187079 (PMC5663428; doi:10.1371/journal.pone.0187079)
Supplement: S1 Table — (DOCX) [file pone.0187079.s001.docx]

**S1 Table. Experimental honey bee groups.**

| **Group No.** | **Sample** | | |
| --- | --- | --- | --- |
|  |  | ***Varroa* mite infestation** | **Thiamethoxam** |
| I, IV | brown-eyed pupa | yes | no |
|  | white-eyed pupa | yes | no |
| II, III | brown-eyed pupa | yes | yes |
|  | white-eyed pupa | yes | yes |
| V, VIII | brown-eyed pupa | no | yes |
|  | white-eyed pupa | no | yes |
| VI, VII | brown-eyed pupa | no | no |
|  | white-eyed pupa | no | no |
