# Supplementary material for: Immune related gene expression in worker honey bee (Apis mellifera carnica) pupae exposed to neonicotinoid thiamethoxam and Varroa mites (Varroa destructor)
Source: PLoS One. 2017 Oct 31;12(10):e0187079. doi: 10.1371/journal.pone.0187079 (PMC5663428; doi:10.1371/journal.pone.0187079)
Supplement: S2 Table — (DOCX) [file pone.0187079.s002.docx]

# S2 Table. Primers used for detection of honey bee pathogens.

| Pathogen | Gene description | F. Primer | R. Primer | Gene ID |
| --- | --- | --- | --- | --- |
| *A.apis* | *Ascosphaera apis* 28S large subunit ribosomal RNA gene | TCTGGCGGCCGGTTAAAGGCTTC | GTTTCAAGACGGGCCACAAAC | AY004344 |
| *A.woodi* | *Acarapis* externus isolate B4E5 cytochrome oxidase subunit I | TCAATTTCAGCCTTTTATTCAAGA | AAAACATAATGAAAATGAGCTACAA | HQ243442.1 |
| ABPV | Acute bee paralysis virus isolate GFf1ab | ACCGACAAAGGGTATGATGC | CTTGAGTTTGCGGTGTTCCT | HM228893.1 |
| BQCV | Black queen cell virus | TTTAGAGCGAATTCGGAAACA | GGCGTACCGATAAAGATGGA | HQ655494.1 |
| DWV | Deformed wing virus isolate | GAGATTGAAGCGCATGAACA | TGAATTCAGTGTCGCCCATA | AY292384.1 |
| IAPV | Israel acute paralysis virus of bees | GCGGAGAATATAAGGCTCAG | CTTGCAAGATAAGAAAGGGGG | EU224279 |
| KBV | Kashmir bee virus | TGAACGTCGACCTATTGAAAAA | TCGATTTTCCATCAAATGAGC | AY275710.1 |
| *N.apis* | *Nosema apis* 16S ribosomal RNA gene | CAATATTTTATTGTTCTGCGAGG | TATATTTATTGTATTGCGCGTGCT | FJ789798.1 |
| *N.ceranae* | *Nosema ceranae* | CAATATTTTATTATTTTGAGAGA | TATATTTATTGTATTGCGCGTGCA | U26533.1 |
| *P.larvae* | *Paenibacillus larvae* | CGGGAGATGAGAAAACCAAT | CCGCAATCGTAAGCTGGTAT | DQ811780.1 |
