# Supplementary material for: Immune related gene expression in worker honey bee (Apis mellifera carnica) pupae exposed to neonicotinoid thiamethoxam and Varroa mites (Varroa destructor)
Source: PLoS One. 2017 Oct 31;12(10):e0187079. doi: 10.1371/journal.pone.0187079 (PMC5663428; doi:10.1371/journal.pone.0187079)

**S1 Fig. Deformed Wings Virus RNA loads in white- and brown-eyed pupae.** Box-plots represents qPCR treshold cycle (CT) values for pathogen loads for different treatments in white-eyed (WE) and brown-eyed (BE) pupae. Treatments: control; thiamethoxam (Thia); Varroa; thiamethoxam and Varroa (Varroa and Thia). In each group (white-eyed pupae and brown-eyed pupae) means with different letters were considered to be statistically significant if the p-value was equal to or less than 0.05.


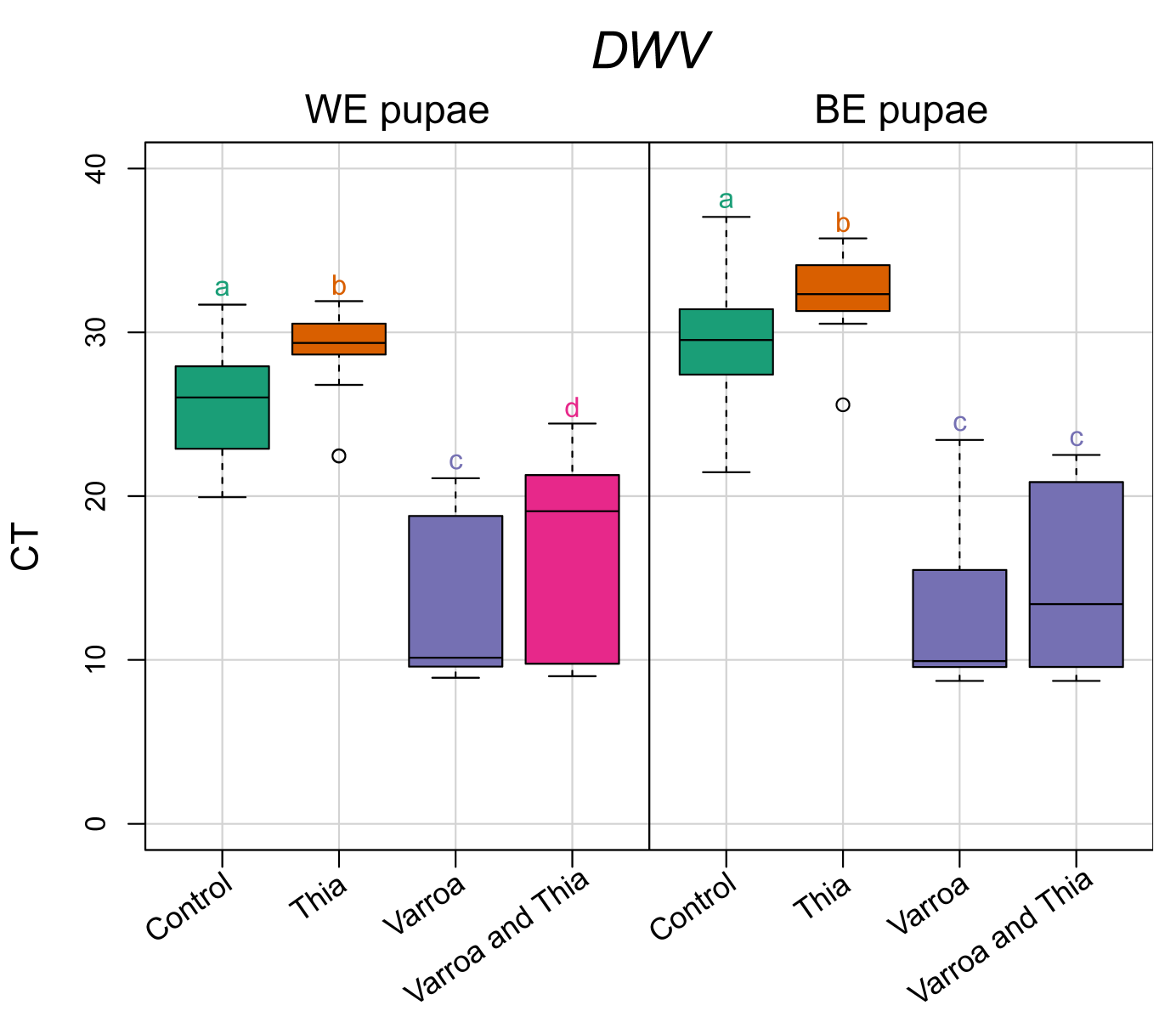

Supplement: S1 Fig — Box-plots represents qPCR treshold cycle (CT) values for pathogen loads for different treatments in white-eyed (WE) and brown-eyed (BE) pupae. Treatments: control; thiamethoxam (Thia); Varroa; thiamethoxam and Varroa (Varroa and Thia). In each group (white-eyed pupae and brown-eyed pupae) means with different letters were considered to be statistically significant if the p-value was equal to or less than 0.05. (DOCX) [file pone.0187079.s003.docx]
