# Supplementary material for: Immune related gene expression in worker honey bee (Apis mellifera carnica) pupae exposed to neonicotinoid thiamethoxam and Varroa mites (Varroa destructor)
Source: PLoS One. 2017 Oct 31;12(10):e0187079. doi: 10.1371/journal.pone.0187079 (PMC5663428; doi:10.1371/journal.pone.0187079)

**S2 Fig. Heatmap analysis of the differences in the mean expression of each gene between Varroa infested and Varroa infested - thiamethoxam treated group**. WE pupae, white-eyed pupae; BE pupae, brown-eyed pupae. The colors indicate the average mRNA levels in Varroa infested - thiamethoxam treated group compared to average levels of mRNA in Varroa infested group: blue indicate lower and red higher levels of expression. Range of relative expression ratio is indicated in the legend on the right.


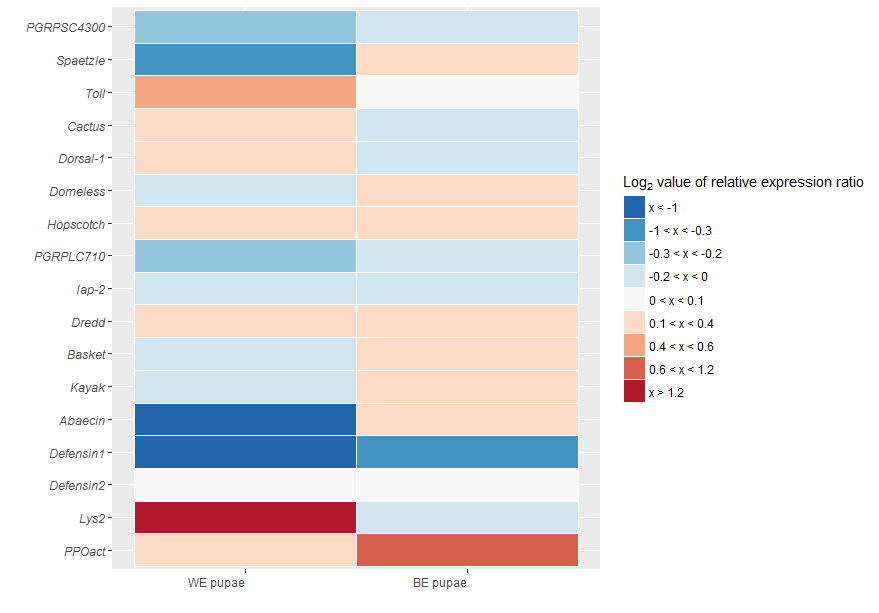

Supplement: S2 Fig — WE pupae, white-eyed pupae; BE pupae, brown-eyed pupae. The colors indicate the average mRNA levels in Varroa infested—thiamethoxam treated group compared to average levels of mRNA in Varroa infested group: blue indicate lower and red higher levels of expression. Range of relative expression ratio is indicated in the legend on the right. (DOCX) [file pone.0187079.s004.docx]
